# The Pathology and Treatment of Gonorrhea

**Published:** 1894-08

**Authors:** Charles M. Blackford

**Affiliations:** Lynchburg, Va.; Fellow Virginia Medical Society, Member Virginia State Medical Examining Board, Secretary Lynchburg Academy of Medicine


### THE PATHOLOGY AND TREATMENT OF GONORRHEA.

By CHARLES M. BLACKFORD, JR., M.D., LYNCHBURG, VA.,

Fellow Virginia Medical Society, Member Virginia State Medical Examining Board, Secretary Lynchburg Academy of Medicine.

Gonorrhea is an acute infectious disease, generally seen in the urethra in the male and the vagina in the female. It is characterized by a productive inflammation, leading to the production of serum and pus, when acute, and to a formation of new connective tissue when chronic.

As stated above, gonorrhea is usually an affection of the genital tract, and, though marked by the same general features in both sexes, the difference in seat produces some differences in the remote effects. Before considering the pathology, it will therefore be necessary to understand fully the anatomical and histological structure of the urethra and vagina.

The male urethra consists essentially of a tube of mucous membrane supported by a submucous layer, which is composed of muscular tissue in the prostatic portion and of fibrous tissue in the spongy portion, but in this latter there is also found a layer of erectile tissue. The urethra being erectile, that is, being of varying length and diameter under different circumstances, its mucous lining is "too large," and is thrown into folds in the flaccid state, so that strictly speaking there is no lumen to the tube in its ordinary condition. The existence of these folds must be borne in mind, as a serious pathological condition is dependent on them.

The urethra is not a simple tube of uniform caliber. The various dilations and contractions should be well known before attempting operations of any kind, even one so simple as the passage of a catheter. The histology of the vagina is simple. It consists of a tube lined with epithelium, and surrounded by muscular coats. In it, as in the urethra, there are openings for gland ducts, each of which may become a source of infection.

This is but a slight glance at the anatomy of these parts, but it is sufficient for our purpose. We pass at once to the symptoms and pathology of the disease. After a brief period of incubation the preliminary symptoms make their appearance. These are tingling and burning in the urethra and at the meatus, increased by passing urine, and, on inspection of the meatus, its lips are seen to be everted and swollen. A serous discharge commences which becomes purulent, and the penis is painful and sore to the touch. The amount of discharge varies in individual cases, and may be slight or very profuse. After a period which may be a few days or may extend over weeks, the discharge may subside and the patient get well, or the disease may pass into a chronic condition known as gleet.

On microscopic examination we find evidence of inflammation. The epithelium lining the canal is swollen, cloudy and degenerated. In places the degenerated cells have been thrown off, and small erosions are visible through the endoscopic tube. Within and between the cells are diplococci, and the same organisms are found in the pus cells. The submucous layers are infiltrated with serum, leucocytes, and, should the case be one of severity, pus may be formed below the mucous membrane. Should the patient recover after a case of short duration, the exuded serum will be absorbed and leave no trace of its existence, but should the exudate organize the folds of mucous membrane will be glued together and a diminution of the lumen at that point result, producing what is called a stricture. It is necessary to understand this point fully to have a clear conception of the pathology of gleet. A stricture is not merely a scar. It is formed beneath the mucous membrane, and is the result of the organization of the exudate. It is evident that the rugæ thus agglutinated form crypts which furnish hiding places for the gonococci, almost beyond the reach of antiseptic agents.

The specific cause of gonorrhea is a diplococcus to which the name of gonococcus has been given. It may be stained by any method and is readily differentiated from other cocci by the fact that by Gram's method, all other cocci found in the urethra will retain the stain, while the gonococci are decolorized. It lies imbedded in the substance of the pus cells, and is consequently difficult to reach, except by first using some agent to decompose the pus cell. As stated above it lies below the surface of the mucous membrane, and is liable to be carried off in the lymph current, and to start secondary abscesses in neighboring glands.

Until very recent times, gonorrheal rheumatism, that disagreeable sequel that follows some cases, was inexplicable, and even at the present day its pathology is far from certain. The study of the ptomaïnes and leucomaïnes, so carefully wrought out by Vaughan and Novy has given us a hypothetical pathology, which further investigation may confirm. It is more than possible that this complication may be the result of the absorption of some poison elaborated by the micro-organism, and that it is preventable by the destruction of the cocci. The failure that has attended efforts to cultivate the micro-organism outside of the body has impeded experiment in this direction, but when this has been overcome it will be possible to test the effects of these by-products. It is held by some that the rheumatism is the result of urinary absorption from the denuded urethra, and the advocates of this view point to the comparative rarity of this complication in women, and claim that whenever it occurs in them that the urethra, if not the bladder also, is involved. This hardly seems to hold good, for were it so the rheumatism would follow any lesion of the urinary lining, which we know is not the case.

When gonorrhea passes into the chronic state it is called gleet. Few subjects in the domain of pathology have been so misunderstood as this, nor have many been more fully explained by modern investigation. The statement made above as to the mode of formation of a stricture must be remembered. A stricture is formed by the fastening together of the rugæ in the mucous membrane and its subjacent tissues by the organization of the lymph poured out in and around them. This forms pockets in which the secretion from the membrane is retained, and in which the gonococci can thrive unmolested. The caliber of the stricture has no effect, for however slight the diminution of the normal lumen be, the sacculation of the membrane is present, and so long as this persists, and so long as the bacteria can live in the pockets so long will the gleet remain.

A stricture opposes a mechanical obstruction to the flow of urine, and behind it some of the urinary sediment will be deposited. This is composed of desquamated cells from the bladder and deep urethra, mucous, and perhaps pus. This *débris* furnishes a moist, warm breeding place for gonococci, and owing to the covering of muco-pus, the position is almost impregnable.

It is this specific nature of the disease that makes the great difference between it and the other forms of urethritis and catarrh in other organs. The treatment of gonorrhea differs from that of other catarrhal affections on account of its seat, and the impossibility of giving functional rest to the part. It is a tractable disease when properly treated, and the most annoying that we encounter when such is not the case.

In the female the lesions are similar to those of the male, though the larger size of the canal renders the disease more easy of access, and diminishes the danger of its becoming chronic. The same danger of stricture exists, but this is not so great as in the male. The lurking place for the disease is the cul-de-sac behind the neck of the uterus, and a knowledge of this fact will go far towards obviating its danger.

The treatment of gonorrhea is a matter of importance to every physician, for like the poor, we have it with us always. A few points must be borne in mind. The first is that we have a narrow tube filled with a muco-purulent secretion, not readily miscible with water, and behind this liquid plug is a sphincter muscle closing the channel. If an injection be made it will but play on the end of this plug, and will not affect the diseased portion of the urethra. Again, this secretion is actively contagious, and if forced into the bladder will extend the disease to that organ. It is therefore necessary to remove the secretion from behind, and treat a clean membrane.

This may be done in several ways. If the patient be told to urinate immediately before the administration of the treatment the urethra will be fairly clean, but irrigation is far better. A small flexible catheter should be lubricated with glycerine and introduced as far as the triangular ligament. It is important to use the glycerine, for any oil will leave a waterproof coating on the urethra and defeat the desired object. Through this catheter about a quart of warm solution of sodium bicarbonate (ten per cent.) should be run, allowing it to return outside the catheter so as to wash the sides of the urethra. The pus will be dissolved by the alkaline wash and will be entirely removed. By raising the fountain syringe any desired pressure may be obtained, and it is well to use enough pressure to dilate the tube so that the follicles may be well washed.

When the water returns clear and free from "tripper faden," the antiseptic wash should be substituted. In my own practice, I use the following:

℞ Hydrarg. chlorid. corros. .......................................... gr. $\frac{1}{3}$.
Sodium chlorid ....................................................... gr. v.
M. Sig. Dissolve in one pint of warm water.

Still using a fountain syringe, I attach a Lindenschmidt dilating irrigator to its tube, and run the whole pint through the urethra bathing its clean walls thoroughly. Using this treatment three times daily, I find the inflammation to subside, the secretion diminish and the case at an end in nine or ten days, as a rule.

I cannot insist too much on the importance of the alkaline wash. Without it the sublimate is useless, for in most cases the antiseptic will not reach the membrane at all, or if it does, it will be precipitated by the albumen in the secretion.

The treatment of gleet may be summarized in three words; cut the stricture. Until that be removed, all means will be useless, for it furnishes a constant source of irritation. It may be destroyed by dilation, or still better by cutting. After this, under antiseptic irrigation, the gleet will rapidly disappear. This statement must be somewhat qualified. Every long contined discharge from the urethra is not a gleet. After an attack of gonorrhea, the mucous membrane may remain in an atonic condition for some time, during which there is a glairy discharge, but this has no tripper-faden nor gonococci. In these cases all treatment directed to the urethra should be stopped, and the general health of the patient improved. Tonics, surf-bathing, or other exercise, will be of advantage. Of course, excessive exertion of any kind must be discouraged, and sexual rest insisted on. Steel sounds cautiously used will often aid recovery, whether by obliterating strictures in course of formation or by some pressure effect on the membrane, I am unable to say.

Experience teaches the sad lesson that there is no treatment that is unfailing, but in a fairly large practice among such cases, I have found that alkaline irrigation, followed by weak antiseptics, gives more satisfactory results than any method in common use.

A word as to internal medication. Except the alkaline diuretics and laxatives, internal medication is of little use. The employment of balsams is hallowed by long usage, and patients expect them, but aside from the mental effect, little or no good will follow them. Rest, alkaline washes, and antisepsis, are the great pillars on which we rely.

